# Changes in Alcohol Consumption and Determinants of Excessive Drinking During the COVID-19 Lockdown in the Slovak Republic

**DOI:** 10.3389/fpubh.2021.791077

**Published:** 2022-02-01

**Authors:** Beata Gavurova, Samer Khouri, Viera Ivankova, Matus Kubak

**Affiliations:** ^1^Institute of Earth Resources, Faculty of Mining, Ecology, Process Control and Geotechnologies, Technical University of Košice, Košice, Slovakia; ^2^Department of Regional Sciences and Management, Faculty of Economics, Technical University of Košice, Košice, Slovakia

**Keywords:** alcohol consumption, drinking, unhealthy behavior, determinants, individual characteristics, substance use, COVID-19 lockdown, Slovakia

## Abstract

As a result of the coronavirus disease 2019 (COVID-19) pandemic, countries have been forced to adopt strong restrictions, such as lockdown, which can lead to serious consequences for public health, including the problematic use of addictive substances. The aim of this cross-sectional study was to examine changes in alcohol consumption and to identify determinants against the background of excessive drinking during the COVID-19 lockdown in the Slovak Republic. The research included 445 respondents (33% males and 67% females), and the data collection through the questionnaire took place from April 29, 2020 to July 1, 2020. Measures such as drinking frequency, amount of alcohol and excessive drinking were used to examine alcohol consumption. Descriptive analysis and binary logistic regression were used to meet the main aim. The findings provide a closer look at the situation in the Slovak Republic and contribute to comprehensive international knowledge. The frequency of excessive drinking did not change in about half of respondents (53% of males and 69% of females). More respondents decreased their excessive drinking than increased, both among males (31 and 16%, respectively) and females (25 and 6%, respectively). Similar results were found for drinking frequency and amount of alcohol. Amongst Slovak respondents, an increase in excessive drinking was more common among males, younger people, smokers, and smokers who increased smoking during the lockdown. Especially in the case of vulnerable populations, public policies should consider a response to impending problems. The findings of this study encourage the implementation of effective and evidence-based prevention programs, which are more than necessary in the Slovak Republic.

## Introduction

The world has been hit by severe acute respiratory syndrome coronavirus-2 (SARS-CoV-2), which has led to hitherto unknown conditions in people's lives. In this context, many countries have established strict measures that have been reflected in social life. These measures were associated with restrictions, quarantine and isolation aimed at defeating the coronavirus disease 2019 (COVID-19) pandemic. It was not possible to observe another situation in the Slovak Republic. People began to face threats such as unknown disease, job loss, limitations in education and opportunities, but also economic recession, and all these aspects could lead to risky behavior (1). Thus, the COVID-19 pandemic can be characterized as a mass trauma with health consequences, including addictive problems. This fact was emphasized by Da et al. (2), who found that social isolation can lead to psychological decompensation and increased alcohol consumption or relapse. The increase in alcohol relapse, but also newly diagnosed individuals with alcohol use disorder and alcohol-related liver disease, may be a result of the phenomena of social isolation. Also, Rehm et al. (3) noted that monitoring alcohol consumption levels during and after the COVID-19 pandemic is necessary to better understand the effects of COVID-19 on alcohol abuse. These facts were the greatest motivation for an international team of researchers who decided at the beginning of the pandemic to examine patterns of unhealthy behavior in 22 European countries. This spectacular effort revealed valuable findings in a sample of 40,064 respondents (4–6), and the presented study is a part of this European research effort.

Increasing alcohol consumption and alcohol-related negative consequences for human health were a major problem in the Slovak Republic even before the pandemic (7). This is evidenced by the fact that the Slovak Republic is one of the countries with the highest levels of alcohol consumption in Europe, while the reason may be easier accessibility of alcoholic beverages, especially during social events and entertainment (7, 8). Alcohol consumption in the Slovak Republic is and has been a common habit of everyday life, while alcohol not only has a calming and relaxing effect on Slovaks, but also causes social and health suffering (9). In this context, the Slovak population in the pre-pandemic period was at risk of alcohol-related diseases and mortality across regions, as well as gender and age groups (10, 11). Different levels of normative (descriptive and injunctive) beliefs prevailed in the Slovak population, influencing the relationship between attitude toward alcohol consumption and individual frequency of alcohol consumption (12). Brutovska et al. (12) revealed that a more positive attitude toward alcohol use, male gender and higher income were associated with more frequent alcohol consumption among young Slovak adults. For these reasons, it is desirable to know a situation in this country during the COVID-19 pandemic. At this point, it should be noted that the lack of scientific and political attention is being paid to the alcohol problems in this country. In addition, similar research has not yet taken place during the pandemic in the Slovak Republic.

In any case, there is evidence from China that during the outbreak of severe acute respiratory syndrome (SARS) in 2003, the symptoms of alcohol abuse and addiction in healthcare workers were associated with quarantine and work in a high-risk location, with people using alcohol as a coping strategy (13). On this basis, there is a need to monitor drinking behavior as well as its determinants against the background of the current COVID-19 pandemic in each country, including the Slovak Republic. In general, excessive drinking is well examined in many countries, but there is a lack of scientific studies in the Slovak Republic addressing this serious problem. Changes in alcohol-related behaviors are inconsistent across countries and their populations, with some individuals reporting an increase in alcohol consumption and others reporting a decrease. Therefore, it is justified to investigate this problem.

The COVID-19 pandemic is an unprecedented phenomenon that requires research in different areas of people's lives in individual countries, including alcohol-related problems. To successfully overcome the consequences of the pandemic, it is essential to know the current situation and the main determinants of health risk behavior in society, as well as vulnerable population groups. This allows for early monitoring and addressing of possible future problems in the field of addictology. For these reasons, the presented study focuses on changes in alcohol consumption and determinants of excessive drinking during the COVID-19 lockdown in the Slovak Republic, filling a research gap in this country. Understanding the problem is especially important for the development of successful strategies and programs aimed at reducing alcohol consumption. Despite the fact that alcohol consumption and its determinants is a well-examined problem around the world, the Slovak Republic is a country that has long neglected and overlooked this threat in society. Insufficiency can be observed not only in the research area, but also at the level of implementation of prevention policies targeted at reducing alcohol consumption in the population. This is reflected in the lack of evidence-based interventions. That is why research is needed in this region. Understanding the situation is especially important during and after the COVID-19 pandemic for the development of successful strategies and programs. The study provides a valuable platform for information on changes in the frequency of alcohol consumption and significant determinants of excessive drinking, which are a key pillar of decision-making by Slovak leaders in the field of public health. Without sufficient evidence, it is not possible to design and implement effective programs that are lacking in this country.

## Materials and Methods

This study is attributed to the efforts of an international research team from 22 European countries to investigate alcohol-related behavioral patterns at the beginning of the COVID-19 pandemic. The survey was conducted in English and was translated into different languages through an international network of researchers. This project was largely covered by the commitment of the European Study Group on Alcohol use and COVID-19. In total, 40,064 respondents were included in the international survey and it is an honor of the authors of this study to present findings from the Slovak Republic. The study contributes to the valuable findings of studies already conducted based on these unique international data (4–6).

### Research Aim and Questions

The findings presented in the introduction underline the importance of examining various aspects of excessive alcohol consumption during the COVID-19 pandemic, as public policy makers in each country should know the evidence-based information for implementing effective strategies. Therefore, the aim of this study was to examine changes in alcohol consumption and to identify determinants against the background of excessive drinking during the COVID-19 lockdown in the Slovak Republic. Following this aim, two research questions were formulated:

- RQ1: Did the Slovak respondents report changes in alcohol consumption during the COVID-19 lockdown?
- RQ2: What are the determinants of excessive alcohol consumption during the COVID-19 lockdown in the Slovak Republic?

### Data

**Figure 1:**
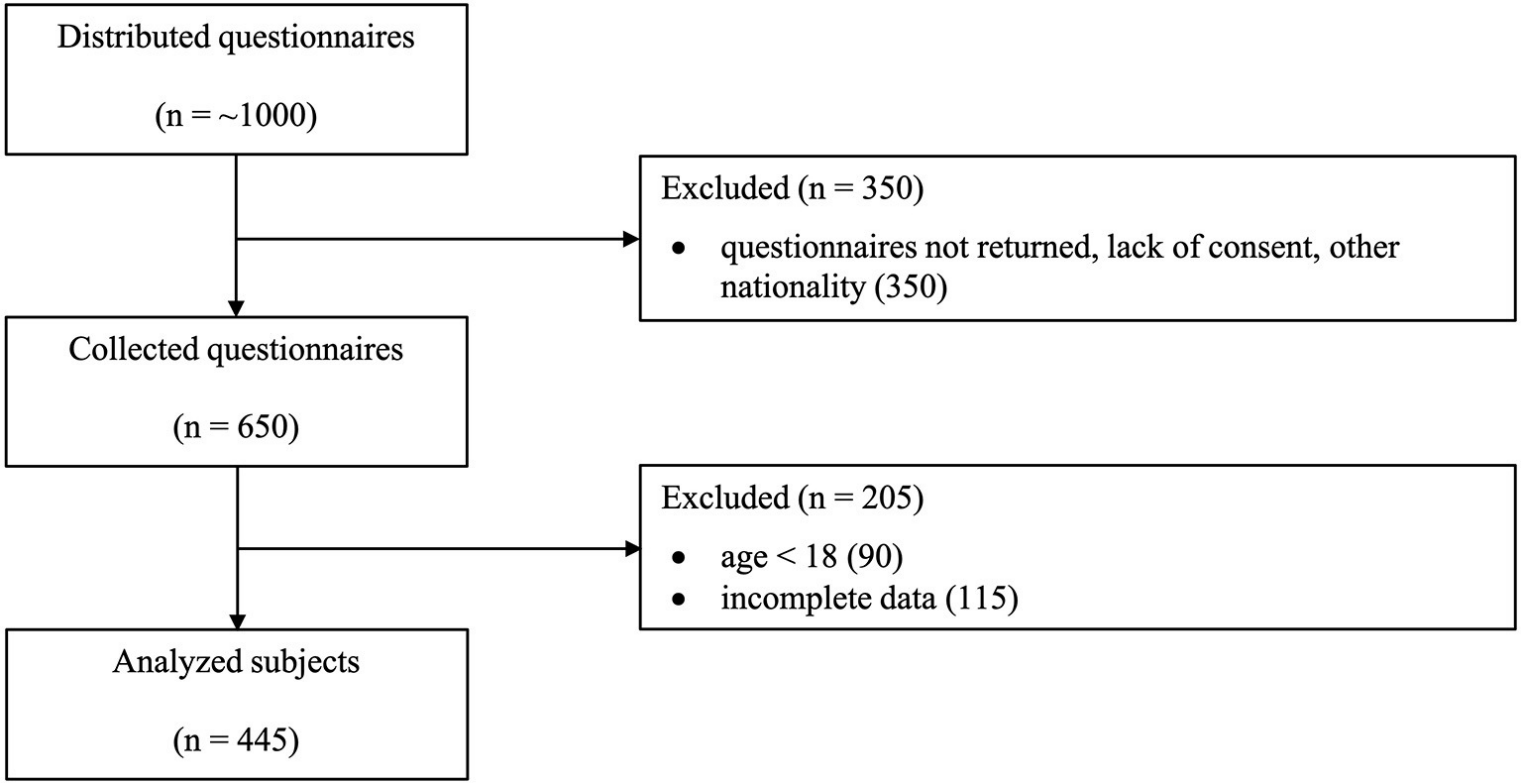
Selection process of respondents to obtain the research sample.

Data were collected using an online questionnaire in 22 European countries in collaboration with a group of international researchers. The questionnaire aimed to reveal information about the relationship between the COVID-19 pandemic and changes in consumption of tobacco, alcohol, cannabis and other illegal drugs in European countries. The questionnaire also collected various socio-demographic information about respondents. This study presents only data for the Slovak Republic, which were collected during the lockdown in the Slovak Republic, which started in the second week of March 2020. Data for the Slovak Republic were collected between April 29, 2020 and July 1, 2020, thus more than 1 month after the outbreak and at the end of the first wave. The respondents were contacted on social networks, and the second form of data collection was direct messages and e-mails requesting the completion of the questionnaire. The respondents did not receive any financial reward and no paid promotion was used. A total of 445 respondents were included in the final research sample. The process of selecting respondents to obtain the final research sample is shown in Figure 1. The selection criteria were approved consent, Slovak nationality, age over 18 years and complete responses.

As indicated, the questionnaire focused on detection of changes in alcohol, tobacco and illegal drug use. However, in this study, attention was focused on changes in alcohol consumption during the lockdown in the Slovak Republic. The research included three questions from the questionnaire, which were proposed by the European team and formulated in the same way for all countries. The questions were formulated as follows:

- Did you drink alcohol less or more often in the past month?
- Did the amount of alcohol you usually drink on each drinking occasion change in the past month?
- Did the frequency of drinking occasions where you drank a high amount of alcohol (i.e., 6 or more drinks) change in the past month?

Based on the initial European effort, the first question focused on changes in drinking frequency, the second question focused on changes in amount of alcohol, and the third question focused on extreme rate of drinking behavior during the lockdown. These three questions offered possible responses using the Likert scale as follows: 1–much less, 2–slightly less, 3–no change, 4–slightly more, 5–much more.

### Governance and Ethics

The survey was completely anonymous and personal data were protected in accordance with the European Union Regulation 2016/679 of the European Parliament and Council, which facilitates ethical assessment. The research was approved by the ethics committee of the Clinical Trials Services, USP TECHNICOM, Technical University of Košice, Slovakia (Ref. 02/04/2021 IG Bioinformatics). All respondents included in this study received the same information about the research and confirmed their informed consent at the beginning of the questionnaire. The respondents did not receive any financial reward. All aspects in this research were conducted with respect to the seventh revision of the World Medical Association–Declaration of Helsinki (14) and the second revision of the Farmington Consensus (15).

This research was supported by the Scientific Grant Agency of the Ministry of Education, Science, Research, and Sport of the Slovak Republic and the Slovak Academy Sciences as part of the research project: VEGA 1/0797/20. Also, this research was supported by the Internal Grant Agency of FaME Tomas Bata University in Zlin: RO/2020/05.

### Statistical Approach

In order to meet the main aim of this study and to answer the research questions, the following statistical approach was chosen. First, a descriptive analysis was provided to present the distribution of the respondents' answers according to gender classification, as well as to identify changes in drinking during the COVID-19 lockdown in the Slovak Republic. This also helps to take a first look at the data. Subsequently, a binary logistic regression was used, in which the dependent variable was the answer to the third question “Did the frequency of drinking occasions where you drank a high amount of alcohol (i.e., 6 or more drinks) change in the past month?” This question was predefined and designed by the European research team led by J. Rehm and was considered an extreme rate of alcohol drinking during the lockdown. Strict measures and interventions were established during the lockdown to defeat COVID-19, and it was therefore interesting to examine the selected question. This provided findings on excessive drinking in the context of the COVID-19 pandemic and related interventions, especially in public life. Other questions from the questionnaire were also examined and the findings will be published separately. Excessive alcohol drinking is a harmful behavior for physical (16) and mental health and is considered a serious social problem, especially in critical situations, such as the COVID-19 pandemic. Adrian and Barry (17) showed that individuals with excessive drinking have a higher morbidity rate for mental disorders. Skinner and Allen (18) found that alcohol abuse has social consequences, even though alcohol is a socially acceptable drug, and is associated with thinking disorders, anxiety, depression, as well as physical symptoms of the cardiovascular, nervous, and digestive systems. As there were five possible answers and the logistic regression uses only a dichotomous variable, the answers “slightly more” and “much more” were included in the first category called “MORE,” and the answers “slightly less,” “much less” and “no change” were included in the second category called “LESS + NO CHANGE.”

The main objective of the performed analysis was to observe changes in alcohol consumption, considering various socio-demographic characteristics of individuals. Backward stepwise regression was used, meaning that the estimation in this study started with all potential explanatory variables in the model, assuming that they could have an impact on excessive drinking during the COVID-19 lockdown. Specifically, the following variables were initially considered in the regression: gender, age, income, changes in income, education, residence, household size, changes in public life caused by the spread of COVID-19, changes in private life caused by the spread of COVID-19, negative consequences in occupational or financial situation due to the spread of COVID-19, smoking, changes in smoking behavior, changes in cannabis use, changes in illegal substance use. The categorization used for these potential explanatory variables is provided in Supplementary Table 1. Subsequently, statistically insignificant variables were sequentially dropped from the model in order to obtain a parsimonious model with only statistically significant variables. Changes in Log Likelihood were used as threshold selection criteria for backward elimination.

Binary logistic regression overcomes restrictive assumptions of linear regression. When using binary logistic regression, the dependent variable does not have to come from a normal distribution. Furthermore, this method does not require a linear relationship between the dependent variable and the regressors. Even if the residues must be independent, they do not have to be distributed normally. The only assumption that needs to be met is the assumption of the absence of multicollinearity among explanatory variables, which was fulfilled in this study.

In the regression model, the examination was focused on the occurrence of the frequency of drinking occasions where the respondent drank a high amount of alcohol (i.e., 6 or more drinks) during the lockdown due to COVID-19, thus the occurrence of excessive alcohol consumption. The regression model formula was as follows:

$$\begin{array}{l} \ln\left(\frac{\mathrm{Pr}\left(excessive\;drinking=MORE\right)}{\mathrm{Pr}\left(excessive\;drining=LESS+NO\;CHANGE\right)}\right)\\ \;=\beta_{0}+\overset{n}{\underset{i=1}{\sum }}\beta_{i}\times x_{i} \end{array}$$

where ln (excessive drinking = MORE) describes a probability with which the dependent variable is “MORE,” while a probability of “LESS+NO CHANGE” value is: Pr (excessive drinking = LESS+NO CHANGE) = 1–Pr (excessive drinking = MORE). The expression *ln*$\left(\frac{Pr\left(excessive\;drinking=MORE\right)\;}{Pr\left(excessive\;drining=LESS+NO\;CHANGE\right)\;}\right)$ is marked as odds or a probability that a respondent drank excessively to a probability that respondent has not and its logarithm is marked as logit. β_0_ is a constant in the model, β_*i*_ denotes the estimated regression coefficients, and *x*_*i*_ is the set of explanatory variables described above. Mathematical editing of the expression of the first equation above resulted in allocation probability to the first group, thus excessive drinking=MORE; and its equation has the following form:

$$\begin{array}{l} Pr\left(excessive\;drinking=MORE\right)\;=\frac{1}{1+e^{-\left(\beta_{0}+\sum_{i=1}^{n}\beta_{i}\times x_{i}\;\right)}} \end{array}$$

As mentioned above, the dependent variable was excessive drinking of alcohol during the COVID-19 lockdown. The dependent variable acquired a value of 1 if the frequency of drinking occasions where the respondent drank a high amount of alcohol (i.e., 6 or more drinks) increased and a value of 0 if it remained unchanged or decreased.

The analytical processing was performed in SPSS v. 19 (IBM, Inc., Armonk, NY, US).

## Results

This section presents the main results with their interpretations and is divided into two subsections according to the analysis used in the research (descriptive analysis, regression analysis).

### Descriptive Analysis

As mentioned above, a total of 445 respondents participated in this research in the Slovak Republic, of which 147 were males and 298 were females (33% males and 67% females). Regarding the profile of respondents, females predominated over males. In terms of age variable, the sample was well equilibrated. Despite the benefits of online surveys, its limitations need to be considered. In this context, young people are much more online and the chance of being reached by a survey is higher. Also, females are more willing to participate in online research. There are also doubts about the capture of alcohol consumption in socially excluded groups (homeless people). These aspects have been addressed and explained in other studies focusing on online surveys of alcohol consumption across the population (19, 20). In this way, the value of the knowledge provided in the presented research is preserved.

The counts of responses are shown in Tables 1–**3**. The first glance at the tables indicated that females' drinking was more stable than males' drinking. Thus, in the case of females, the answer “no change” was selected much more often than in the case of males.

**Table 1:** Distribution of answers: frequency of drinking.

		**Gender**			
		**Males**		**Females**	
		**Count**	**Percentage**	**Count**	**Percentage**
Did you drink alcohol less or more often in the past month?	Much less	34	23.1%	56	18.8%
	Slightly less	17	11.6%	32	10.7%
	No change	60	40.8%	169	56.7%
	Slightly more	25	17.0%	34	11.4%
	Much more	11	7.5%	7	2.3%

Table 1 shows the answers to the first question: Did you drink alcohol less or more often in the past month?

Based on the results in Table 1, it can be noted that 56.7% of females and 40.8% of males declared that their frequency of drinking did not change. “Much less” drinking was found in 23.1% of males and 18.8% of females, and “slightly less” in 11.6% of males and 10.7% of females. On the other hand, “slightly more” drinking was reported by 17% of males and 11.4% of females. The option “much more” was chosen by 7.5% of males and 2.3% of females. These results revealed that about half of the respondents did not change their drinking frequency during the first wave of the pandemic. Gender differences indicated that females were more stable than males in their drinking patterns. The changed alcohol consumption among the respondents was reflected in a decrease more than in an increase, for both males and females.

Table 2 shows the distribution of answers on the second question: Did the amount of alcohol you usually drink on each drinking occasion change in the past month?

**Table 2:** Distribution of answers: amount of alcohol.

		**Gender**			
		**Males**		**Females**	
		**Count**	**Percentage**	**Count**	**Percentage**
Did the amount of alcohol you usually drink on each drinking occasion change in the past month?	Much less	29	19.7%	41	13.8%
	Slightly less	18	12.2%	26	8.7%
	No change	74	50.3%	205	68.8%
	Slightly more	20	13.6%	22	7.4%
	Much more	6	4.1%	4	1.3%

As Table 2 shows, in the second analyzed question, it was again possible to observe a relatively large difference between males and females. The answer “no change” was chosen by 68.8% of females and 50.3% of males. The answer “much less” was identified in 19.7% of males and 13.8% of females. The option “slightly less” was chosen by 12.2% of males and 8.7% of females. The “slightly more” option was chosen by 13.6% of males and 7.4% of females and the “much more” option was chosen by 4.1% of males and only 1.3% of females. Based on these results, it could be stated that the amount of alcohol consumed during the early COVID-19 pandemic did not change clearly in half of the respondents. Thus, the onset of the pandemic did not affect alcohol doses in these cases. The observed change was characterized by a lower amount of alcohol rather than a higher one. Again, females were more stable in their drinking patterns and less prone to higher alcohol consumption.

The answers to the third question “Did the frequency of drinking occasions where you drank a high amount of alcohol (i.e., 6 or more drinks) change in the past month” are shown in Table 3.

**Table 3:** Distribution of answers: excessive drinking.

		**Gender**			
		**Males**		**Females**	
		**Count**	**Percentage**	**Count**	**Percentage**
Did the frequency of drinking occasions where you drank a high amount of alcohol (i.e., 6 or more drinks) change in the past month?	Much less	28	19.0%	55	18.5%
	Slightly less	18	12.2%	20	6.7%
	No change	78	53.1%	206	69.1%
	Slightly more	14	9.5%	14	4.7%
	Much more	9	6.1%	3	1.0%

Table 3 presents the results of a descriptive analysis of the collected answers to the last question, which concerns in particular excessive drinking. Gender differences in behavior were also evident in this analyzed case. “No change” in excessive alcohol drinking during the lockdown due to COVID-19 was found in 53.1% of males and 69.1% of females. The option “much less” was selected by 19% of males and 18.5% of females. “Slightly less” excessive drinking was reported by 12.2% of males and 6.7% of females. In the case of increased excessive alcohol consumption during the lockdown, the following results can be identified, 9.5% of males declared “slightly more” frequency of drinking occasions where they drank a high amount of alcohol and the option “much more” was found in 6.1% of male respondents. Females reported “slightly more” frequency of drinking occasions where they drank a high amount of alcohol in 4.7% of the analyzed cases and the option “much more” in 1% of these cases. In general, the results of excessive drinking revealed similar findings as in the previous cases. Thus, the change in excessive drinking during the early COVID-19 pandemic was not identified in more than half of the research sample. Less excessive drinking prevailed over more excessive drinking, while males appeared to be a vulnerable group in terms of changed risk behavior and excessive drinking.

### Regression Analysis

The values of the coefficients of the binary logistic regression model for excessive drinking are presented in Table 4. The suitability of the model as a whole was verified by the Hosmer-Lemeshow test, with a *p*-value of 0.787 (Chi-square = 4.722, with 8 degrees of freedom), thus it could be assumed that the binary logistic regression model was correctly estimated. All possible explanatory variables were first considered, but only gender, age, smoking and changes in smoking behavior proved to be statistically significant variables in terms of an increase in excessive drinking. Thus, the final model in this study included explanatory variables such as gender, age, smoking and changes in smoking behavior:

- *Gender*–a binary categorical variable with a range of values: 0–female, 1–male.
- *Smoking*–a binary categorical variable with a range of values: 0–non-smoker, 1–smoker.
- *Changes in smoking behavior*–a categorical variable obtained as an answer to the question: Did you smoke less or more often in the past month? Permissible values were: 0–no change, 1–less smoking, 2–more smoking.
- *Age*–an interval variable with a range of values: ≤23, 24–27, 28–35, 36–45, 46+. The age variable was binned into intervals so that the individual age groups were equally numerous. Age was divided into quintiles to capture trends within given age groups of respondents, not just to obtain overall measure that would be captured if age was treated as a continuous variable.

**Table 4:** Logistic regression analysis of the odds of an increased frequency of excessive drinking amongst respondents.

		**95% Wald confidence interval for odds ratio**		
	**Odds ratio**	**Lower**	**Upper**	***p*-value**
**Gender**				
Males	2.15	0.97	4.78	0.061
**Smoking**				
Smokers	3.36	1.49	7.57	0.003
**Change in smoking behavior**				
Change in smoking (less)	1.84	0.44	7.73	0.404
Change in smoking (more)	3.40	1.07	10.84	0.038
**Age**				
Age ≤ 23	5.25	1.10	25.06	0.038
Age 24–27	1.50	0.25	8.98	0.660
Age 28–35	5.76	1.14	29.37	0.040
Age 36–45	3.50	0.60	20.52	0.170

*p-value was based on the Wald test. Reference categories: Gender (females); Smoking (non-smokers); Change in smoking behavior (no change). Age (46+)*.

The reference categories for given variables were set as follows:

- *Gender*–“female” gender was set as a reference category because descriptive analysis indicated that males were more prone to increased alcohol consumption.
- *Smoking*–“non-smoker” was set as a reference category because there was a presumption of a possible interdependence between alcohol and tobacco use.
- *Changes in smoking behavior*–“no change” was set as a reference category because the intention was to observe the possible interrelation between changes in smoking and changes in drinking.
- *Age*–“age interval 46+” was set as a reference category due to the assumption that the lockdown negatively affected rather young people than middle-aged people and older people in terms of excessive drinking.

The interpretation of the obtained regression coefficients is as follows. An increase in excessive drinking during the lockdown was more common among males than among females. Males were 2.15 times more likely to increase their excessive drinking than females [95% Confidence Interval (CI): 0.97–4.78]. With a focus on smoking, smokers were 3.36 times more prone to increase excessive drinking during the lockdown than non-smokers (95% CI: 1.49–7.57). In terms of changes in smoking behavior, the contrast value was “no change.” Respondents who reported that they smoked more during the lockdown compared to the usual situation were 3.40 times more likely to increase excessive drinking than smokers whose smoking doses remained unchanged (95% CI: 1.07–10.84). It turns out that people have tendencies to use tobacco and alcohol interdependently, as compared to people who do not smoke. Moreover, an increase in smoking and an increase in excessive alcohol consumption seemed to be interconnected. On the other hand, less smoking was not significant. Focusing on age, where 46+ was a contrast value, the findings were as follows. Respondents aged <23 years had 5.25 times higher chance of increased excessive drinking during the lockdown than respondents aged 46 years and more (95% CI: 1.10–25.06). The results for the age ranges 24–27 and 36–45 years were not statistically significant. Respondents aged 28–35 years were generally 5.76 times more likely to increase their excessive alcohol drinking during the lockdown than respondents aged 46 years and more (95% CI: 1.14–29.37).

## Discussion

The COVID-19 pandemic caused problems in many dimensions of countrie's lives (21). In the health dimension, the serious problems that existed before COVID-19 have not disappeared, but on the contrary, the pandemic exacerbated these problems in many cases. During the pandemic, not only reducing the incidence of COVID-19 (22) but also reducing alcohol consumption remains a public health priority (23).

### Changes in Alcohol Consumption

In this study, most females did not report a change in alcohol consumption in terms of their answers to alcohol-related questions. In the individual analyzed cases, 56.7 to 69% of females reported unchanged drinking, while males, who did not change their drinking, ranged from 40.8 to 53.1%. Thus, males tended to change alcohol use more than females. In general, alcohol consumption in Slovaks did not change in about half of the respondents, which is in line with the previous European studies (6). In a comparison with European team of researchers, their results also showed that alcohol consumption did not change in about half of the respondents from European countries (6). With a focus on changed drinking in this study, it can be concluded that the change was reflected in a decrease in alcohol consumption more than in an increase, for both males and females. This is consistent with the findings revealed in the European study by Manthey et al. (6) and it can be concluded that the Slovak respondents reported similar rates as the European population included in the international research effort. The findings are in line with the assumption that lower levels of alcohol consumption can be expected at the beginning of the pandemic due to reduced alcohol availability (3). This can also be explained by the fact that during the COVID-19 lockdown, the availability of alcohol was lower in terms of closed bars, pubs, cafes, clubs and restaurants, and people could consume alcohol bought in the store only at home (24, 25). The change in off-premises alcohol consumption may not have been substantial in the short term, as could also be seen in an Australian study conducted by Vandenberg et al. (26).

A closer look at the results shows that 34.7% of males and 29.5% of females reported less frequent drinking during the COVID-19 pandemic. On the other hand, 24.5% of males and 13.7% of females reported more frequent drinking. Regarding the amount of alcohol used on drinking occasions, 32.2% of males and 22.4% of females reported lower amounts during the COVID-19 pandemic, while 17.8% of males and 8.6% of females reported higher amounts. Finally, the drinking occasions where the respondents drank a high amount of alcohol, declaring excessive alcohol drinking, were less frequent in 31.2% of males and 25.2% of females during the COVID-19 pandemic. This type of drinking occasions was more frequent in 15.6% of males and 5.7% of females. The results of this study can be compared with many others revealed in different countries. A very similar gender comparison was provided by a study from the UK (27) and it can be concluded that Slovak respondents reported more positive results than UK respondents, especially females. It is also possible to focus on other countries and their outcomes during the COVID-19 pandemic. Chodkiewicz et al. (28) found that more than 30% of respondents from their Polish survey changed their drinking as a result of the COVID-19 pandemic. According to the results, 16% of respondents drank less and 14% drank more. Similar results were revealed by Sidor and Rzymski (29). In France, 24.4% of alcohol drinkers reported a decrease in their alcohol consumption since the lockdown and 10.7% reported an increase (30). In Norway, 29.9% of respondents reported they drank less, whereas 13.3% reported they drank more (31). Thus, the tendency of changes in drinking was similar for respondents from the Slovak Republic as for respondents from other countries, where a decrease in alcohol consumption prevailed compared to an increase. Interestingly, in Germany, 34.7% of respondents reported that they drank more alcohol during the lockdown, 19.4% drank less alcohol, 37.7% reported no changes in their drinking patterns, and 8.2% did not drink alcohol (32). In this way, Slovak respondents reported more positive rates. These facts demonstrate the diversity of alcohol-related behavior across populations, and the determinants of change in alcohol consumption should be examined.

### Determinants of Increased Excessive Drinking

Among other factors, it can be concluded that factors such as male gender, being smoker, more smoking and young age can be associated with an increase in excessive alcohol consumption on drinking occasions during the COVID-19 pandemic. Similar findings were revealed in other studies (1, 33–36).

The comorbidity of alcohol and tobacco consumption seems to be very important finding in terms of addressing substance use behavior. This indicates a comorbidity between various unhealthy behaviors, specifically smoking and drinking. In this context, it is possible to agree with the findings of Reynolds et al. (37), who revealed that increases in alcohol consumption were associated with increases in tobacco use during the COVID-19 lockdown. Similar findings were revealed by Zadarko-Domaradzka et al. (38), who focused on young adults in the Carpathian Euroregion. This finding can be explained by the fact that one type of risk behavior may encourage individuals to engage in other risk behaviors. Accordingly, the association between risk alcohol consumption and the use of other addictive substances was also confirmed in other studies (39–41).

This study also contributes to the knowledge that young age is considered a determinant of increased alcohol consumption, as confirmed by Vanderbruggen et al. (36), Calina et al. (1) and Gonçalves et al. (33). Also, Jacob et al. (35) emphasized that a higher proportion of respondents with increased alcohol consumption was more pronounced among young adults aged 18–34 years. Similar results can be found in other studies (42). For instance, the study of Ahmed et al. (43) revealed that young adults aged 21–40 years were more vulnerable in terms of their mental health conditions and alcohol consumption during the COVID-19 pandemic. The explanation can be found in social, enhancement and coping motives, which are strong at a young age (44, 45). In this context, alcohol expectancies can play an important role in this fact, and it is also true that expectancies linearly decrease with increasing age (46). This encourages the implementation of campaigns to inform the population about the potential long-term effects of increased alcohol and tobacco consumption (47).

From a gender point of view, other studies have also shown that males are prone to change in drinking, while they tended to drink more than females (33, 34, 42, 43). These findings reflect the well-known fact that males are more vulnerable to drinking than females (12, 48, 49). Males are characterized by less healthy lifestyles, less social and normative barriers, and they also perceive alcohol-related problems differently than females; therefore, fewer protective factors can be expected for males (48, 50). This can also be seen in the light that, although females generally consume less alcohol than males, it is females who suffer a much higher risk of alcohol-related health damage and present a more vulnerable profile with less willingness to undergo treatment (49, 51). On the other hand, male vulnerability was not confirmed in a study conducted by Garnett et al. (52).

### Implications for Public Policies

It is important to realize that alcohol is already a long-term problem in society, which represents a burden from a health, economic and social point of view (53–55), while the COVID-19 pandemic is another serious threatening factor (1, 56–58). In the Slovak Republic, addiction and alcohol abuse were ubiquitous diseases even before the pandemic, but it is the pandemic that can make them worse. For this reason, Slovak experts in the field of addictology can be expected to face major challenges even after the pandemic. All these facts underline the need to monitor changes in drinking among populations during and after the COVID-19 pandemic (59, 60).

The findings of this study should be considered when developing prevention programs aimed to reduce risky drinking in the Slovak Republic, where these programs are lacking. This study is an important appeal for policy-makers and decision-makers to make greater efforts to improve the situation, to pay increased attention to this problem, to remove barriers and to create adequate conditions. Monitoring alcohol consumption across the Slovak population is essential, but it is also very important to put effective measures into practice and compare the results. Alcohol-related problems should be integrated into general health policies, strategies and interventions. It is public health policies that have the potential to influence alcohol consumption in the population (3, 61). Based on the presented results, it is recommended that Slovak decision-makers and policy-makers develop alcohol use prevention policies for younger individuals, males and smokers. Regarding the identified reduction in alcohol consumption during the COVID-19 lockdown in Slovaks, there are opportunities to encourage people to continue this trend, as well as to motivate individuals with plans to quit drinking and reduce their alcohol consumption. The implementation of public measures and interventions to stop drinking forever without being exposed to stigma seems to be an effective step (62, 63). Campaigns and programs aimed at reducing alcohol consumption, eliminating misinformation and health education are even more justified in the case of vulnerable populations. Slovak public policies and services should consider a response to impending problems and harmful drinking not only during the COVID-19 pandemic, but also in the post-pandemic period. Improving health literacy across the Slovak population can help to achieve positive outcomes in the field of health and addictology (64, 65), and there are other effective measures (66).

### Strengths, Limitations and Future Directions

The strengths of the study lie in its importance and value for the country, where this social problem has long been overlooked and unresolved, resulting in inadequate services and interventions to help addicts. This study contributes with important findings on the change in alcohol consumption and determinants against the background of excessive drinking during the COVID-19 lockdown in the Slovak Republic. The presented results offer a closer look at the situation in the Slovak Republic and contribute to comprehensive international knowledge. The findings of this study encourage the implementation of effective and evidence-based strategies, which are more than necessary in this country. A valuable platform of results revealed by the presented research can help this effort regardless of the pandemic period. The pandemic multiplies the power of research findings and requires that all tools and information be used to successfully address this sensitive and very often stigmatized social problem.

This research did not avoid the limitations to which the partially unbalanced nature of the research sample could be included. Thus, there was a higher proportion of females and younger respondents. However, this deficiency should not be considered as undermining the value of knowledge in the Slovak Republic. Another possible limitation is the fact that the study included a smaller research sample that was investigated only in one country.

By respecting these limitations, future research should include a larger sample and more countries should be involved in the research. Also, future research ambitions should be focused on examining the individual characteristics linked to changes in unhealthy behavior on a larger scale. Accordingly, other patterns of unhealthy behavior (tobacco use, illegal drug use, excessive use of sedatives, non-substance use) and other individual factors (having children, living situation, type of employment, work status, marital status, education level, mental health) should be taken into account and covered in future research. Personal perceptions of health, self-care and self-monitoring could offer a more detailed look at changes in population behavior during the COVID-19 pandemic. Last but not least, future research should focus on investigating the problem after the COVID-19 pandemic, with an emphasis on established interventions and measures.

## Conclusions

The COVID-19 pandemic disrupted people's lives in many dimensions, which could translate into changes in unhealthy behavior, including alcohol consumption. For public health professionals and leaders, it is important to know the current situation and the main determinants of excessive alcohol drinking. This was provided for Slovak professionals and leaders in the presented study with the ambition of further investigation.

The aim of this study was to examine changes in alcohol consumption and to identify determinants against the background of excessive drinking during the COVID-19 lockdown in the Slovak Republic. Descriptive analysis and binary logistic regression were used to meet the main aim. The main findings showed that the frequency of excessive drinking did not change in about half of respondents. With a focus on changed excessive drinking, it was possible to conclude that the change was reflected in a decrease more than in an increase, both among males and females. Amongst Slovak respondents, an increase in excessive drinking was more common among males, younger people, smokers, and smokers who increased smoking during the lockdown. In fact, the individual characteristics linked to alcohol consumption need to be constantly examined, not only in the short term but also in the long term, as the consequences of the pandemic can last for a long time. The authors appeal to alcohol prevention with a closer focus on young adults, males and current smokers, as they seem to be most at risk from problematic alcohol consumption.

## Data Availability Statement

The original contributions presented in the study are included in the article/Supplementary Material, further inquiries can be directed to the corresponding author/s.

## Ethics Statement

The study was conducted according to the guidelines of the Declaration of Helsinki. The survey was completely anonymous and personal data were protected in accordance with the European Union Regulation 2016/679 of the European Parliament and Council, which facilitates ethical assessment. The research was approved by the Ethics Committee of the Clinical Trials Services, USP TECHNICOM, Technical University of Košice, Slovakia (Ref. 02/04/2020 IG Bioinformatics). Informed consent was obtained from all subjects involved in the research.

## Author Contributions

BG: conceptualization, investigation, resources, writing—original draft preparation, writing—review and editing, supervision, project administration, and funding acquisition. SK: conceptualization, investigation, writing—original draft preparation, writing—review and editing, supervision, project administration, and funding acquisition. VI: conceptualization, investigation, resources, writing—original draft preparation, writing—review and editing, visualization, and supervision. MK: conceptualization, methodology, software, validation, formal analysis, investigation, writing—original draft preparation, and writing—review and editing. All authors contributed to manuscript revision, read, and approved the submitted version.

## Funding

This research was funded by the Scientific Grant Agency of the Ministry of Education, Science, Research, and Sport of the Slovak Republic and the Slovak Academy Sciences as part of the research project VEGA 1/0797/20: Quantification of Environmental Burden Impacts of the Slovak Regions on Health, Social and Economic System of the Slovak Republic. This research was supported by the Internal Grant Agency of FaME Tomas Bata University in Zlin: RO/2020/05.

## Conflict of Interest

The authors declare that the research was conducted in the absence of any commercial or financial relationships that could be construed as a potential conflict of interest.

## Publisher's Note

All claims expressed in this article are solely those of the authors and do not necessarily represent those of their affiliated organizations, or those of the publisher, the editors and the reviewers. Any product that may be evaluated in this article, or claim that may be made by its manufacturer, is not guaranteed or endorsed by the publisher.
