# Supplementary material for: Changes in Alcohol Consumption and Determinants of Excessive Drinking During the COVID-19 Lockdown in the Slovak Republic
Source: Front Public Health. 2022 Feb 1;9:791077. doi: 10.3389/fpubh.2021.791077 (PMC8843933; doi:10.3389/fpubh.2021.791077)
Supplement: Supplementary file 1 [file Table_1.docx]

Supplementary Material

**Supplementary Table 1.** All potential explanatory variables initially considered in the regression model

| **Explanatory variable** | **Categories** |
| --- | --- |
| **Gender:**   - a binary categorical variable obtained as an answer to the questionnaire item: Please specify your gender. | 0 – female (*reference category*)  1 – male |
| **Age:**   - an interval variable obtained as an answer to the questionnaire item: Please specify your age. | ≤23  24–27  28–35  36–45  46+ (*reference category*) |
| **Income:**   - an interval variable obtained as an answer to the questionnaire item: Please specify your monthly net household income before the outbreak of SARS-CoV-2. | ≤499 EUR (*reference category*)  500–999 EUR  1,000–1,499 EUR  1,500–1,999 EUR  2,000–2,499 EUR  2,500–2,999 EUR  3,000–3,499 EUR  3,500–3,999 EUR  4,000–4,499 EUR  4,500+ EUR |
| **Changes in income:**   - a categorical variable obtained as an answer to the questionnaire item: Please specify the change in your monthly net household income since the outbreak of SARS-CoV-2 in your country. | 0 – no change (*reference category*)  1 – strong increase (more than 50% increase)  2 – moderate increase (25%–50% increase)  3 – small increase (below 25% increase)  4 – small decrease (below 25% decrease)  5 – moderate decrease (25%–50% decrease)  6 – strong decrease (more than 50% decrease) |
| **Education:**   - a categorical variable obtained as an answer to the question: What is the highest school grade you have completed? | 0 – less than high school (*reference category*)  1 – high school  2 – any education beyond high school |
| **Residence:**   - a categorical variable obtained as an answer to the question: Which of these categories best describes the type of place where your main residence is located? | 0 – village/farm (*reference category*)  1 – small city or town  2 – medium-size city  3 – large city |
| **Household size:**   - an interval variable obtained as an answer to the question: How many people are permanently living in your household, including yourself? | 0 – 1 person (*reference category*)  1 – 2–3 persons  2 – 4–5 persons  3 – 6+ persons |
| **Changes in public life caused by the spread of COVID-19:**   - a categorical variable derived from the question: In the past month, did you perceive any restrictions of public life, which were implemented to contain the spread of SARS-CoV-2? | 0 – not at all (*reference category*)  1 – to some degree  2 – to a substantial degree  3 – to a very high degree |
| **Changes in private life caused by the spread of COVID-19:**   - a categorical variable derived from the question: In the past month, did you experience any restrictions of your everyday life as a result of measures implemented to contain the spread of SARS-CoV-2? | 0 – not at all (*reference category*)  1 – to some degree  2 – to a substantial degree  3 – to a very high degree |
| **Negative consequences in occupational or financial situation due to the spread of COVID-19:**   - a categorical variable derived from the question: In the past month, have you experienced any negative consequences concerning your occupational or financial situation in relation to the spread of SARS-CoV-2? | 0 – not at all (*reference category*)  1 – to some degree  2 – to a substantial degree  3 – to a very high degree |
| **Smoking:**   - a binary categorical variable derived from the questionnaire item on change in smoking. | 0 – non-smoker (*reference category*)  1 – smoker |
| **Changes in smoking behavior:**   - a categorical variable obtained as an answer to the question: Did you smoke less or more often in the past month? | 0 – no change (*reference category*)  1 – less  2 – more |
| **Changes in cannabis use:**   - a categorical variable obtained as an answer to the question: Did you consume cannabis less or more often in the past month? | 0 – do not consume (*reference category*)  1 – much less  2 – slightly less  3 – no change  4 – slightly more  5 – much more |
| **Changes in illegal substance use:**   - a categorical variable obtained as an answer to the question: Did you consume illegal drugs (except cannabis) less or more often in the past month? | 0 – do not consume (*reference category*)  1 – much less  2 – slightly less  3 – no change  4 – slightly more  5 – much more |
